# Mechanical convergence in mixed populations of mammalian epithelial cells

**DOI:** 10.1140/epje/s10189-024-00415-w

**Published:** 2024-03-27

**Authors:** Estelle Gauquelin, Keisuke Kuromiya, Toshinori Namba, Keisuke Ikawa, Yasuyuki Fujita, Shuji Ishihara, Kaoru Sugimura

**Affiliations:** 1 Department of Biological Sciences, Graduate School of Science, The University of Tokyo, Tokyo, 113-0032 Japan; 2 Department of Molecular Oncology, Graduate School of Medicine, Kyoto University, Kyoto, 606-8501 Japan; 3 Universal Biology Institute, The University of Tokyo, Tokyo, 113-0033 Japan; 4 Graduate School of Arts and Sciences, The University of Tokyo, Tokyo, 153-0041 Japan; 5 Division of Biological Science, Graduate School of Science, Nagoya University, Aichi, 464-8602 Japan; 6 Department of Computational Biology and Medical Sciences, Graduate School of Frontier Sciences, The University of Tokyo, Chiba, 277-8561 Japan

## Abstract

**Abstract:**

Tissues consist of cells with different molecular and/or mechanical properties. Measuring the forces and stresses in mixed-cell populations is essential for understanding the mechanisms by which tissue development, homeostasis, and disease emerge from the cooperation of distinct cell types. However, many previous studies have primarily focused their mechanical measurements on dissociated cells or aggregates of a single-cell type, leaving the mechanics of mixed-cell populations largely unexplored. In the present study, we aimed to elucidate the influence of interactions between different cell types on cell mechanics by conducting in situ mechanical measurements on a monolayer of mammalian epithelial cells. Our findings revealed that while individual cell types displayed varying magnitudes of traction and intercellular stress before mixing, these mechanical values shifted in the mixed monolayer, becoming nearly indistinguishable between the cell types. Moreover, by analyzing a mixed-phase model of active tissues, we identified physical conditions under which such mechanical convergence is induced. Overall, the present study underscores the importance of in situ mechanical measurements in mixed-cell populations to deepen our understanding of the mechanics of multicellular systems.

**Graphical abstract:**

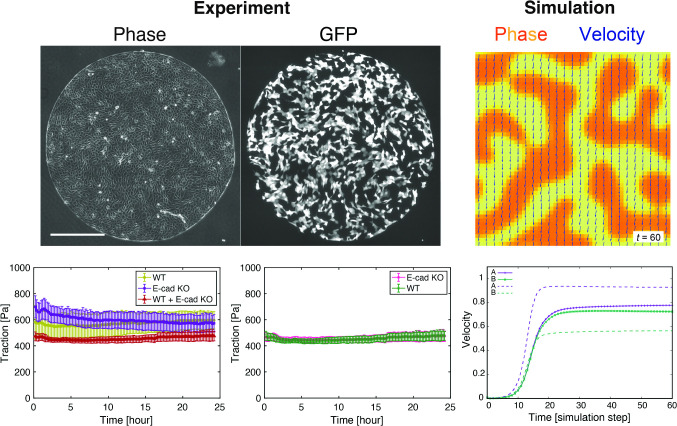

**Supplementary Information:**

The online version contains supplementary material available at 10.1140/epje/s10189-024-00415-w.

## Introduction

Tissues in health and disease are composed of cells with different molecular and/or mechanical characteristics. The development, homeostasis, and dysfunction of tissues arise from cell-cell communications within such heterotypic tissue environments [1–7].

Distinct cell types within a tissue communicate through mechanical forces as well as biochemical signaling [2, 4, 8–13]. For instance, stress transmission from one cell type to another can induce the remodeling of the extracellular matrix [14]. The precise formation of compartment boundaries relies on the differences in cell adhesion strength [15, 16]. Forces between oncogenic cells and their neighboring normal cells determine which will be eliminated from the tissue, a process driven by short-range cell–cell communications known as cell competition [17–19]. Identifying the mechanisms through which mechanical communication between diverse cell types controls tissue dynamics will deepen our understanding of the physics of multicellular systems.

To address this, it is crucial to measure the forces and stresses in mixed-cell populations. In many previous studies, mechanical measurements have focused on dissociated cells or aggregates of a single-cell type [20–24]. This can be attributed, in part, to the challenges associated with *in situ* measurements of intercellular stress, which were difficult to achieve until recent advancements [25, 26]. Often, results from single-cell-type measurements have been used to interpret the dynamics of mixed-cell populations under the assumption that cell mechanics remain consistent regardless of the presence of other cell types. However, this assumption has not been rigorously tested, leaving the mechanics of mixed-cell populations largely unexplored.

Bayesian inversion stress microscopy (BISM) offers a mean to measure intercellular stress within a monolayer of cultured cells [27]. Traction force microscopy (TFM) is used to measure the traction exerted by the cells on a substrate [28]. BISM then computes the stress tensor from the measured traction. This method has been validated using synthetic data and successfully utilized to characterize stress-dependent cellular behaviors such as apoptosis and rigidity sensing [27, 29–32]. Using BISM, we recently quantified intercellular stress under conditions that initiate cell competition between oncogenic cells and their surrounding normal counterparts [33]. Our findings indicate that normal cells decreased the isotropic component of intercellular stress when co-cultured with oncogenic RasV12 cells. This alteration in isotropic intercellular stress subsequently triggers downstream biochemical signaling, leading to the apical extrusion of RasV12 cells. These findings highlight the significance of conducting mechanical measurements directly within mixed-cell populations to shed light on the mechanical regulation of tissue homeostasis. However, our previous study did not analyze the isotropic stress in RasV12 cells. Moreover, it remains to be determined whether other mechanical quantities, such as deviatoric stress and cell velocity, undergo changes similar to isotropic stress during cell competition.

In the present study, we aimed to elucidate the influence of the interactions between different cell types on epithelial mechanics. Taking the advantage of BISM, we conducted *in situ* mechanical measurements in mixed populations of wild-type and genetically modified Madin–Darby canine kidney (MDCK) cells. Our results demonstrated that although individual cell types exhibited distinct levels of traction and intercellular stress before mixing, in the mixed monolayer, these mechanical values were nearly identical between the cell types. This convergence of traction and intercellular stress occurs in the context of both cell competition and differential cell adhesion. Finally, by analyzing a mixed-phase model of active tissues, we showed that the degree of phase separation influences whether two cell types, each with inherently distinct mechanical properties, merge into a single cohesive entity upon mixing.

## Results

To study the cell mechanics in mixed populations, we conducted *in situ* mechanical measurements in a monolayer of cultured epithelial cells. TFM [28] and BISM [27] were employed to quantify the traction exerted by cells on the substrate and the stress tensors within the monolayer (Materials and Methods).

The cells exert tractions $\overrightarrow{T}(x,y,t)~=~[T_x(x,y,t),T_y(x,y,t)]$ on the substrate. Over each time frame for each monolayer, we calculated the total magnitude of the force $|F(t)|~=~\int \int (T_x^2(x,y,t)+T_y^2(x,y,t))^{1/2}~\textrm{d}x\textrm{d}y$. Subsequently, the value |*F*(*t*)| was averaged between experiments performed with the same cell lines in order to obtain traction values plotted over time. Using BISM, we obtained the intercellular stress tensor $$\sigma = \bigl ( {\begin{matrix} \sigma _{xx} &{} \sigma _{xy} \\ \sigma _{xy} &{} \sigma _{yy} \end{matrix}} \bigr )$$, which is symmetrical, through the relationship div $\sigma = \overrightarrow{T}$. The isotropic stress $\sigma _{iso}$ is a scalar value calculated by taking the trace of the stress tensor $\sigma$ as $\sigma _{iso} = \frac{\sigma _{xx}+\sigma _{yy}}{2}$. It is the opposite of the pressure inside the tissue, meaning that negative values of $\sigma _{iso}$ represent compression in the tissue, and positive values indicate that the tissue is under tension. The deviatoric stress $\sigma _{dev}$ is a tensor defined as $\sigma _{dev} = \sigma - \sigma _{iso}I$ where *I* is the identity matrix. As $\sigma _{dev}$ is a 2-D symmetrical and traceless matrix, it possesses two eigenvalues, $\lambda _{dev}$ and $-\lambda _{dev}$, that have the same magnitude but opposite signs. We adopted $|\lambda _{dev}|$ as the magnitude of the deviatoric stress. Similar to the traction values, the magnitudes of isotropic and deviatoric stresses were averaged first over space and then over experiments.

**Fig. 1:**
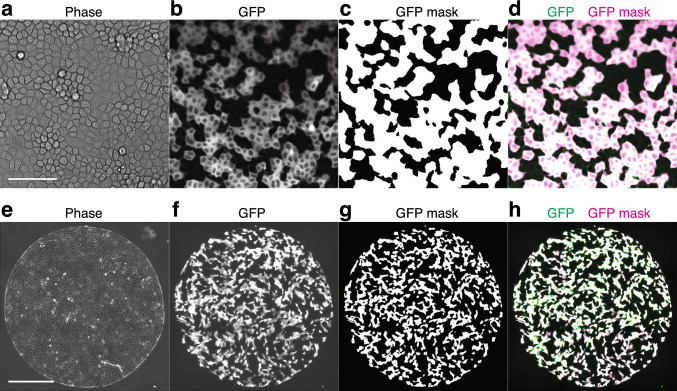
Comparison between GFP fluorescent signal images and their corresponding GFP masks of mixed monolayers. (**a**–**d**) Comparison for a WT+RasV12 monolayer comprising GCaMP WT MDCK cells and Myc-tagged RasV12-induced MDCK cells. Phase contrast (**a**), GFP (GCaMP) (**b**), GFP mask (**c**), and the overlay of the GFP image (green) with the GFP mask (magenta) (**d**) at 6 h after the induction of RasV12 expression. (**e**–**h**) Comparison for a WT+E-cad KO monolayer comprising GFP WT MDCK-II cells and non-tagged E-cad KO MDCK-II cells. Phase contrast (**e**), GFP (**f**), GFP mask (**g**), and the overlay of the GFP image (green) with the GFP mask (magenta) (**h**) at 24 h after cell seeding. Scale bars: 100 ${\upmu }$m (**a**) and 500 ${\upmu }$m (**e**)

The experiments were performed using either a single-cell line or a mixture of two cell lines (Fig. 1). By comparing the mechanical behaviors of cells in each type of experiment, we aimed to elucidate how the interactions between different cell types influence cell mechanics.

### WT cells and RasV12 cells, respectively, decreased and increased traction and intercellular stress in the presence of the other, converging to comparable levels

In multicellular tissues, cells compete for limited resources, such as space and growth factors [34, 35]. Depending on differences in cellular fitness, this cell competition can lead to the elimination of unwanted cells, including those with reduced differentiation potential or those harboring oncogenic mutations [6, 36].

To assess traction and intercellular stress during the early phase of cell competition between normal and oncogenic cells, we mixed wild-type (WT) MDCK cells with RasV12 inducible MDCK cells at a 1:1 ratio and seeded them on collagen-coated soft substrates (Fig. 1a–d; Materials and Methods) [33]. We conducted the mechanical measurement at 6–10 hour (h) after the induction of RasV12 expression. Because the extrusion of RasV12 cells starts at considerably later stages [33, 37], both the WT and RasV12 cells remained in a cohesive monolayer during this period. Throughout the duration of our measurement, the number of cells in the monolayers increased only by around 10%. The change in the cell density was thus negligible.

**Fig. 2:**
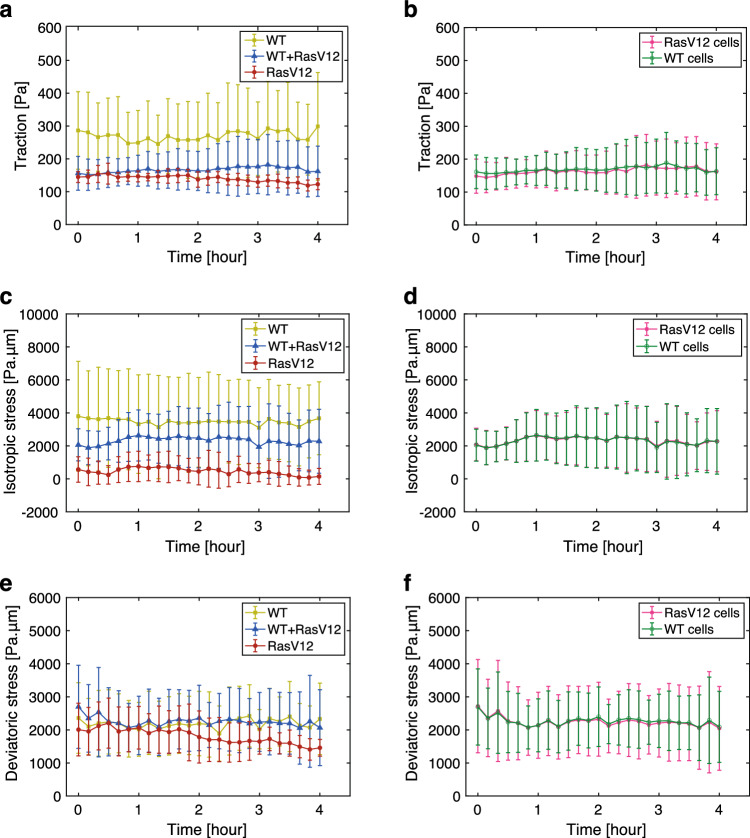
**Quantification of traction, isotropic stress, and deviatoric stress during the early phase of cell competition in MDCK cells.** **a**, **b** Magnitude of the traction exerted by the cells in a function of time, depending on the experiment types (**a**) and on the cell types within the WT+RasV12 co-culture experiments (**b**) ($n = 3$ for each plot). 0 h corresponds to 6 h after the induction of RasV12 expression. In (**a**), WT represents the co-culture of GCaMP WT and non-tagged WT MDCK cells (light green), WT+RasV12 represents the co-culture of GCaMP WT and Myc-tagged RasV12-induced MDCK cells (blue), and RasV12 represents the co-culture of CMFDA-stained RasV12-induced and unstained RasV12 MDCK cells (red). In (**b**), the traction magnitude of each cell type (WT: green, RasV12: magenta) in the WT+RasV12 co-culture experiments is plotted. **c**, **d** Isotropic stress within the monolayers in a function of time, depending on the experiment type (**c**) and on the cell type within the WT+RasV12 co-culture experiments (**d**), as shown in (**a**) and (**b**). **e**, **f** Magnitude of deviatoric stress within the monolayers in a function of time, depending on the experiment type (**e**) and on the cell type within the WT+RasV12 co-culture experiments (**f**), as shown in (**a**) and (**b**). Two-sample *t* test: WT vs. RasV12, WT vs. Mix, and RasV12 vs. Mix, p < 0.001 (**a**); WT vs. RasV12, WT vs. Mix, and RasV12 vs. Mix, $p < 0.001$ (**c**); WT vs. RasV12, $p < 0.001$, WT vs. Mix, $p > 0.1$, RasV12 vs. Mix, $p < 0.001$ (**e**). Data are presented as the mean ± standard deviation (s.d.)

The WT cells exerted higher traction to their substrate and exhibited larger isotropic and deviatoric stresses over time than the RasV12-induced cells when cultured separately (light green and red lines in Fig. 2a, c, e; Video A1). In the monolayer of a mixture of WT and RasV12-induced cells (hereafter referred to as the WT+RasV12 monolayer), the values of traction and isotropic stress fell between those in the WT and RasV12 monolayers (blue lines in Fig. 2a, c; Video A1). The magnitude of deviatoric stress was comparable between the WT and WT+RasV12 monolayers (light green and blue lines in Fig. 2e). We also verified that the variance in mechanical quantities observed across the different types of experiments was not influenced by the size of the region of interest (ROI) (Fig. A1a, c, e, g).

In the analysis described above, the mechanical quantities were averaged over the whole monolayer without distinguishing between cell types. When we analyzed the data from the WT+RasV12 monolayer to differentiate between WT and RasV12 cells, both the traction and intercellular stress levels were closely aligned between the two cell types (green and magenta lines in Fig. 2b, d, f). These data clearly indicated that co-culturing WT and RasV12 cells induced a shift in traction and intercellular stress, leading them to converge to comparable levels.

### Calcium sparks in WT cells did not alter traction and intercellular stress in neighboring cells

**Fig. 3:**
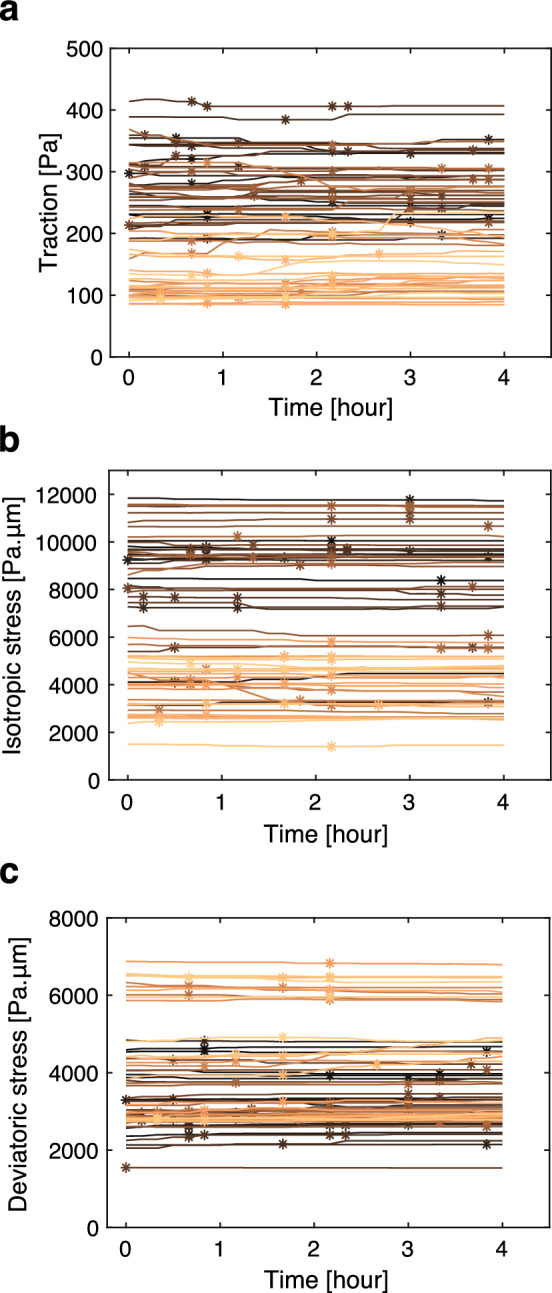
Traction and intercellular stress remain unchanged before and after calcium sparks in MDCK cells. **a**–**c** Quantification of traction (**a**), isotropic stress (**b**), and deviatoric stress (**c**) values near cells exhibiting calcium sparks in the WT+RasV12 monolayer comprising GCaMP WT MDCK cells and Myc-tagged RasV12-induced MDCK cells. The horizontal axis indicates time after the start of image acquisition, with the 0-hour mark corresponding to 6 h after induction of RasV12 expression. Measurements were made within a 53 ${\upmu }$m radius surrounding cells exhibiting calcium sparks. Lines represent data for each ROI. Calcium spark occurrences are indicated by stars

A decrease in isotropic intercellular stress in WT cells induces transient upsurges of intracellular calcium, called calcium sparks [33]. To address the potential changes in cell mechanics following calcium sparks, we tracked the traction and intercellular stress over time in regions proximal to the cells producing calcium sparks. Figure 3 shows no significant trend in the evolution of any mechanical quantity in the area surrounding the cells undergoing a calcium spark event (ROI radius: d = 53 ${\upmu }$m in Fig. 3, $d = 29 {\upmu }$m and $d = 102 {\upmu }$m in Fig. A2). These results exclude the possibility of a feedback mechanism from calcium sparks to the traction and intercellular stress.

### Convergence of traction and intercellular stress in co-culture of other cell types

Next, we investigated whether the convergence of traction and intercellular stress upon co-culturing different cell lines can be observed in other cell combinations. For this, we turned to MDCK-II cells, which are known to exhibit a more spread morphology and higher motility than MDCK cells [38]. Mechanical measurements were conducted as in MDCK cells, except that MDCK-II cells adhered to soft substrates through fibronectin and were confined in 1.8 mm diameter circles (Fig. 1e–h; Materials and Methods) [39]. To eliminate the potential effects of cell proliferation, such as density-dependent jamming [40], we treated the cells with a cell cycle inhibitor, Mitomycin C, before observation (Materials and Methods).

MDCK-II cells with different cell–cell adhesion strengths were examined in this study. Such cell–cell adhesion differences can lead to segregation into domains of distinct cell types [15, 16, 41]. Specifically, we used E-cadherin (E-cad) knockout (KO) cells [42]. The E-cad KO cells maintain a cohesive monolayer through Cadherin-6 [43].

**Fig. 4:**
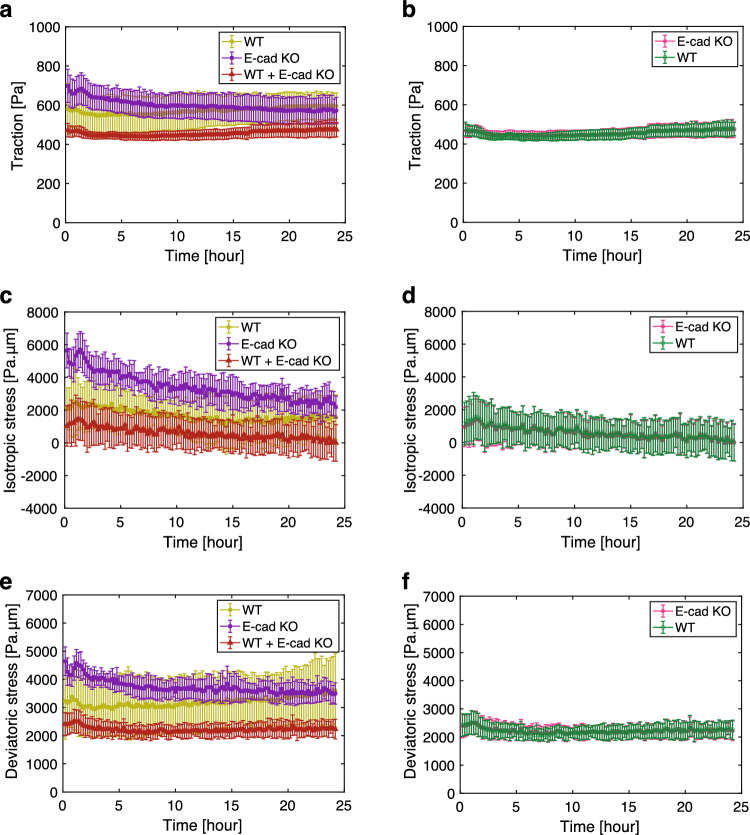
Quantification of traction, isotropic stress and deviatoric stress upon mixing WT and E-cad KO MDCK-II cells. **a**, **b** Magnitude of the traction exerted by the cells in a function of time, depending on the experiment types (**a**) and on the cell types within the WT+ E-cad KO co-culture experiments (**b**) (WT: n = 8, E-cad KO: $n = 6$, and WT+ E-cad KO: $n = 8$). 0 h corresponds to 24 h after the cell seeding. In (**a**), WT represents the WT MDCK-II cells in single-cell colonies (light green), E-cad KO represents the E-cad KO MDCK-II cells in single-cell colonies (purple), and WT+E-cad KO represents the co-culture of WT and E-cad KO MDCK-II cells (red). In (**b**), the traction magnitude of each cell type (WT: green, E-cad KO: magenta) in the WT+E-cad KO co-culture experiments is plotted. **c**, **d** Isotropic stress within the monolayers in a function of time, depending on the experiment type (**c**) and on the cell type within the WT+E-cad KO co-culture experiments (**d**), as shown in (**a**) and (**b**). **e**, **f** Magnitude of deviatoric stress within the monolayers in a function of time, depending on the experiment type (**e**) and on the cell type within the WT+E-cad KO co-culture experiments (**f**), as shown in (**a**) and (**b**). Two-sample *t* test: WT vs. E-cad KO, WT vs. Mix, and E-cad KO vs. Mix, $p < 0.001$ (**a**); WT vs. E-cad KO, WT vs. Mix, and E-cad KO vs. Mix, $p < 0.001$ (**c**); WT vs. E-cad KO, WT vs. Mix, and E-cad KO vs. Mix, $p < 0.001$ (**e**). Data are presented as the mean ± s.d.

TFM analysis confirmed that the traction exerted by the E-cad KO cells was higher than that exerted by the WT cells during the early phase (Fig. 4a), which is consistent with previous studies showing that E-cad KO results in the higher traction and an increase in the size of the focal adhesions [43]. As expected from the elevated traction, the E-cad KO monolayer displayed larger isotropic and deviatoric stresses than the WT monolayer (Fig. 4a, c, e; Video A2). When the WT and E-cad KO cell were co-cultured, the traction and intercellular stress dropped below those observed in the WT monolayer (Fig. 4a, c, e; Video A2). Within the mixed monolayer, both the WT and E-cad KO cells exhibited traction and intercellular stress of the same magnitude, respectively (Fig. 4b, d, f). Similar to the case of cell competition, the size of the ROI did not impact the evolution of mechanical quantities (Fig. A1b, d, f, h). Collectively, these findings suggest that the convergence of traction and intercellular stress in mixed monolayers is a general characteristic of epithelial cell populations and is not specific to the context of cell competition.

### Intercellular stress exhibited no strong dependency on the distance to the cell-type boundaries in co-culture experiments

**Fig. 5:**
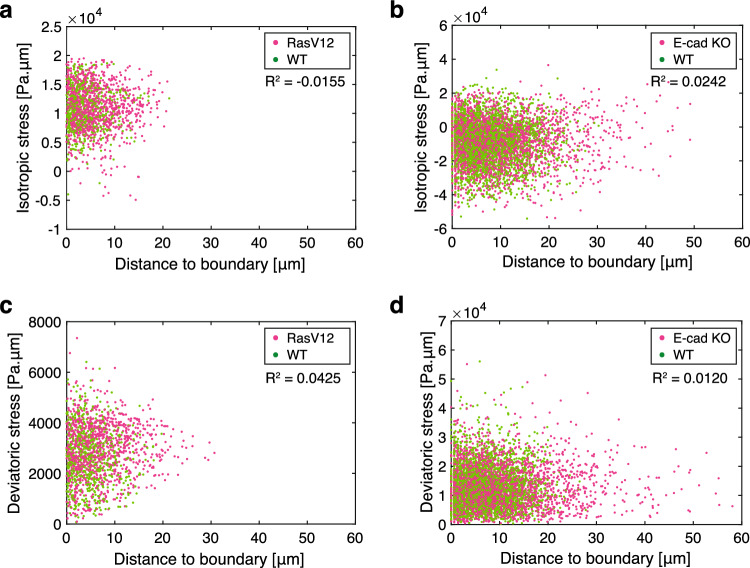
Dependency of intercellular stress magnitude on the distance from the cell-type boundaries in co-culture experiments. **a**–**d** Scatter plots of isotropic stress (**a**, **b**) and deviatoric stress magnitude (**c**, **d**) in co-culture experiments in a function of the distance to the closest boundary between the two mixed-cell types within the monolayer. The correlation coefficient is shown in the top-right corner. **a**, **c** Data obtained at 8 h after the induction of RasV12 expression are plotted. Green and magenta represent WT MDCK cells and RasV12 MDCK cells, respectively. **b**, **d** Data obtained at 42 h after the cell seeding (18 h after the start of image acquisition) are plotted. Green and magenta represent WT MDCK-II cells and E-cad KO MDCK-II cells, respectively

To identify factors contributing to the convergence of intercellular stress upon mixing different cell types, we investigated whether the proximity of one cell type had any effect on the intercellular stress within the other cell types. To this end, we analyzed the correlation between intercellular stress and the shortest distance to cell-type boundaries. We observed no clear dependence on the distance to the cell-type boundaries for either isotropic stress or deviatoric stress magnitude at 8 h after the induction of RasV12 expression in the WT and RasV12 co-culture (Fig. 5a, c), and at 42 h after the cell seeding in the WT and E-cad KO co-culture, respectively (Fig. 5b, d). We also determined that the distance dependency was negligible at earlier time points. In the WT and RasV12 co-cultures, the absolute values of the correlation between the stress magnitude and the distance to the cell-type boundaries ($|R^2|$) were $< 0.02$ at 6 h after the induction of RasV12 expression. In the WT and E-cad KO co-culture, $|R^2|$ was $< 0.03$ at 24 h after the cell seeding. These results show that the change in intercellular stress is not restricted to the cell-type boundaries, but instead suggests the possibility that it is collectively induced within the monolayer through a mechanism that can act over long distances.

### Continuum model for the self-propelled binary cell mixture identifies physical conditions for mechanical convergence

**Fig. 6:**
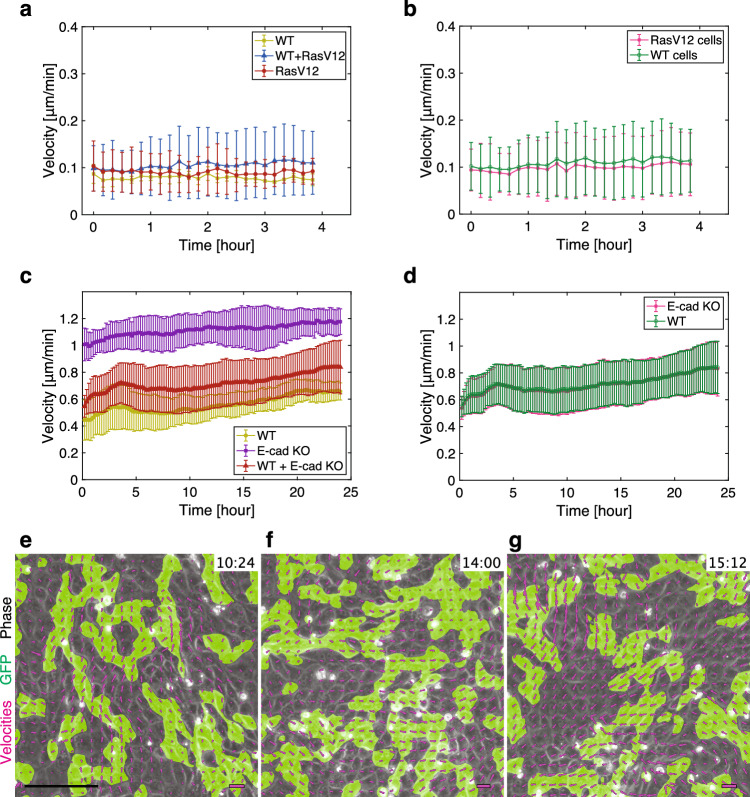
Quantification of velocity magnitudes and orientations in co-culture experiments. **a**, **b** Magnitude of velocity during the early phase of cell competition in within MDCK cell monolayers in a function of time, depending on the experiment types (**a**) and on the cell types within the WT+RasV12 co-culture experiments (**b**) ($n = 3$ for each plot). 0 h corresponds to 6 h after the induction of RasV12 expression. In (**a**), WT represents the co-culture of GCaMP WT and non-tagged WT MDCK cells (light green), WT+RasV12 represents the co-culture of GCaMP WT and Myc-tagged RasV12-induced MDCK cells (blue), and RasV12 represents the co-culture of CMFDA-stained RasV12-induced and unstained RasV12 MDCK cells (red). In (**b**), the velocity magnitude of each cell type (WT: green, RasV12: magenta) in the WT+RasV12 co-culture experiments is plotted. **c**, **d** Magnitude of velocity upon mixing WT and E-cad KO MDCK-II cells in a function of time, depending on the experiment type (**c**) and on the cell type within the WT+E-cad KO co-culture experiments (**d**) (WT: $n = 8$, E-cad KO: $n = 6$, WT+ E-cad KO: $n = 8$). 0 h corresponds to 24 h after the cell seeding. In (**c**), WT represents the WT MDCK-II cells in single-cell colonies (light green), E-cad KO represents the E-cad KO MDCK-II cells in single-cell colonies (purple), and WT+E-cad KO represents the co-culture of WT and E-cad KO MDCK-II cells (red). In (**d**), the velocity magnitude of each cell type (WT: green, E-cad KO: magenta) in the WT+E-cad KO co-culture experiments is plotted. **e**–**g** Phase contrast (gray), GFP mask (green), and velocity field (magenta arrows) at the indicated time in mixed-culture of GFP WT MDCK-II cells and non-tagged E-cad KO MDCK-II cells. To allow for better readability, velocity arrows are scaled to match the grid size with the maximum velocity value at each frame being equal to one unit of grid size. Data in **a**–**d** are presented as the mean ± s.d. Scale bars: 150 ${\upmu }$m (e, lower left), 2 ${\upmu }$m/min (**e**–**g**, lower right)

To elucidate a long-range mechanism driving the mechanical convergence in mixed-cell populations, we introduced a two-dimensional hydrodynamic model for a mixture of two types of active cells [44–47]. In our model, different types of cells inherently have different viscosities and self-propelled forces. This formulation of self-propelled cell populations is motivated by experimental observations showing that different cell types in mixed-cultures moved at the same average speed and in the same orientation, despite exhibiting distinct velocities when cultured separately, a phenomenon particularly evident in MDCK-II cells, which exhibited higher motility than MDCK cells (Fig. 6). In addition, to reflect long-range rotational movement in the MDCK-II monolayer, we introduced a polar variable representing cellular polarity, along which cells move.

We extended the active polar model into a binary mixture, employing an approach similar to that used for the active nematic model in [45, 47]. The model is described by a phase-field variable $\phi ({\varvec{r}},t)$, cellular polarity ${{\varvec{p}}}({\varvec{r}},t)$, and velocity ${\varvec{v}}({\varvec{r}},t)$, where ${\varvec{r}}$ and *t* represent position and time, respectively. The phase field $\phi$ is introduced to represent the binary cell mixture, where $\phi = 1$ indicates the area occupied by cell type A, and $\phi = -1$ denotes the area occupied by cell type B. Free energy associated with these fields is introduced in the generic form as

1 $$\begin{aligned} \begin{aligned} F({\varvec{p}}, \phi )&= \int \left( \frac{a}{4}(1-\phi ^2)^2+\frac{K_\phi }{2}|\nabla \phi |^2 \right. \\&\quad \left. + \frac{a_p}{2}(1-|{\varvec{p}}|^2)^2 + \frac{K_p}{2}|\nabla {\varvec{p}}|^2 \right) \hbox {d}A~ . \end{aligned} \end{aligned}$$

Phase field $\phi ({\varvec{r}},t)$ and polar order ${{\varvec{p}}}({\varvec{r}},t)$ are dimensionless variables, and associated parameters *a*, $K_\phi$, $a_p$, and $K_p$ are all positive (see Table A1 for the list of parameters). The first and second terms promote the phase separation $\phi = \pm 1$ with the interface width $\sqrt{K_\phi /2a}$. The third term promotes polar order with $|{{\varvec{p}}}| = 1$, while the fourth term results in the alignment of the neighboring polar order with the correlation length $\sqrt{K_p/2a_p}$. The time evolution of these fields is given by

2 $$\begin{aligned} \partial _t \phi + v_k \partial _k \phi&= \nabla \left( M \nabla \mu \right) ~, \end{aligned}$$

3 $$\begin{aligned} \partial _t {{\varvec{p}}} + v_k \partial _k {\varvec{p}}&= -\Omega {\varvec{p}} + \kappa D {\varvec{p}} -\Gamma {\varvec{H}}~. \end{aligned}$$

Here, $\mu = \delta F/\delta \phi$ and ${\varvec{H}} = \delta F/\delta {\varvec{p}}$ represent the chemical potential for $\phi$ and molecular field for ${\varvec{p}}$, respectively (Materials and Methods). $D \equiv ({{\varvec{\nabla }} v} + {{\varvec{\nabla }} v}^T)/2$ and $\Omega \equiv ({{\varvec{\nabla }} v} - {{\varvec{\nabla }} v}^T)/2$ are the symmetric and anti-symmetric parts of the velocity gradient tensor. $\kappa$ is a dimensionless parameter for controlling the alignment of the cell polarity under a simple shear flow; we put $\kappa > 0$, indicating ‘rod-like’ in terms of liquid crystal theory, where the cell polarity is enhanced in the direction of shear extension. *M* and $\Gamma$ are parameters to determine the timescale of cell mobility and the cell polarity dynamics.

The cell populations are modeled as viscous Newton fluids, and the velocity field ${{\varvec{v}}}(t, {\varvec{r}})$ is determined by the force balance (Stokes) equation and the incompressible condition. We assumed incompressibility due to the absence of cell division.

4 $$\begin{aligned} \nabla \cdot \left( \nu \nabla {\varvec{v}}\right) - \nabla P + \nabla \cdot \sigma ^e&= -\alpha {\varvec{p}} + \xi {\varvec{v}}~, \end{aligned}$$

5 $$\begin{aligned} \nabla \cdot {\varvec{v}}&= 0~ . \end{aligned}$$

Here, *P* represents pressure, and elastic stress tensor $\sigma ^e$ is derived from the variational of the free energy Eq. 1 (Materials and Methods). Cells interact with their substrate via a self-propelled active force $\alpha {\varvec{p}}$ and friction $- \xi {\varvec{v}}$. In our system, the self-propelled force is the only active force driving the dynamics. As mentioned above, different types of cell populations exhibit distinct velocities. The coefficient of the self-propelled force is set as $\alpha = \alpha _A$ for A cells and $\alpha = \alpha _B$ for B cells, and thus, the intrinsic cell migration speed is given as $\alpha _A \xi ^{-1}$ and $\alpha _B \xi ^{-1}$, respectively. The viscosities of the cells are also different, with the viscous coefficient $\nu = \nu _A$ for A cells and $\nu = \nu _B$ for B cells. Thus, we took

6 $$\begin{aligned} \alpha&= \alpha _A \frac{1+\phi }{2} + \alpha _B \frac{1-\phi }{2}~,~ \end{aligned}$$

7 $$\begin{aligned} \nu&= \nu _A \frac{1+\phi }{2} + \nu _B \frac{1-\phi }{2}~, ~ \end{aligned}$$

where $\alpha _A$ and $\alpha _B$ indicate self-propelled force coefficients of cell type A and B, respectively, etc. We set $\alpha _A = 2\alpha _B$ and $\nu _A < \nu _B$ assuming that A cells are faster and less viscous than B cells.

We numerically solved Eqs. 2–5 on a $50 \times 50$ square region. In the simulation, we set $\alpha _A = 1.0$, $\alpha _B = 0.5$, $\nu _A = 5.0$, $\nu _B = 10.0$, and $\xi = 1.0$. Then, the intrinsic cell migration speed of cell A is two times faster than that of cell B, reflecting the experimental observation in MDCK-II cells (Fig. 6) (see Materials and Methods for the rationale of the parameter choice). The following parameters were also fixed: $a=0.1$, $K_\phi = 0.05$, $\Gamma = 5.0$, $M = 10.0$, $a_p = 0.1$, and $\kappa = 0.7$.

**Fig. 7:**
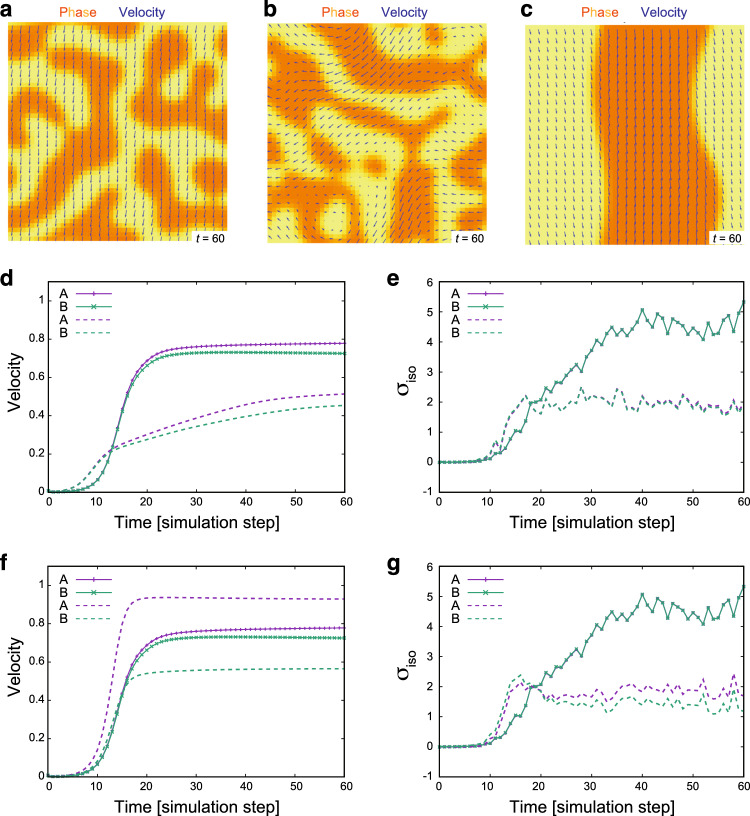
Numerical simulation of binary cell mixture and resulting velocity and stress profiles. **a**–**c** Visualization of the phase field (color scale) and velocity field (blue arrows) at the indicated simulation time. Orange and yellow indicate regions occupied by cells A ($\phi =1$) and cells B ($\phi = -1$), respectively. Parameters for the self-propelled force and viscosity are set as $\alpha _A = 1.0$, $\alpha _B = 0.5$, $\nu _A = 5.0$, and $\nu _B = 10.0$ (see the main text for the definition of parameters). **a** Simulation started from a random initial condition with the polarity alignment parameter set to $K_p = 1.0$. See Video A3 for the full sequence. **b** Simulation started from the random initial condition with $K_p = 2.0 \times 10^{-2}$. See Video A4 for the full sequence. **c** Simulation conducted with the same parameter set as in (**a**), but with a different initial condition that completely segregates the two phases. See Video A5 for the full sequence. **d**, **e** Temporal evolution of the mean velocity (**d**) and $\sigma _{iso} = \frac{\sigma _{xx}+\sigma _{yy}}{2}$ (**e**) for individual cell types (cells A: purple, cells B: green), with solid lines in corresponding to the data from **(a**) and dashed lines to (**b**). Note that purple and green solid lines overlapped in (**e**). **f**, **g** Temporal evolution of the mean velocity (**f**) and $\sigma _{iso} = \frac{\sigma _{xx}+\sigma _{yy}}{2}$ (**g**) for individuals cell types (cells A: purple, cells B: green), with solid lines in corresponding to the data from (**a**) and dashed lines to (**c**). In these simulations, the parameters are set as $\eta = 1.0$, $a=0.1$, $K_\phi =0.05$, $\Gamma = 5.0$, $M =10.0$, $a_p = 0.1$, $\xi =0.7$

Figure 7a, along with Video A3, illustrates an example from our numerical simulation that began with a random initial condition. In the mixture of the two phases, which inherently have a twofold difference in intrinsic velocity, the mean velocities converged to comparable levels (solid lines in Fig. 7d). Similarly, the stress magnitudes became indistinguishable among the two phases (solid lines in Fig. 7e). In a different set of simulations, reducing the coupling of cell polarity led to a more disordered pattern in the velocity field, yet the significant shift in the mean velocity and the isotropic stress was still apparent upon mixing (Fig. 7b and Video A4; dashed lines in Fig. 7d, e). These results demonstrate that the global shift and convergence in the stress and velocity fields can be collectively induced within the mixed-cell population.

Moreover, our numerical simulations revealed that the extent of mechanical convergence was influenced by the degree of phase separation. Using the same parameters as in Fig. 7a but starting from an initial condition of completely segregated phases, each phase retained its distinct velocities and stresses (Fig. 7c and Video A5; dashed lines in Fig. 7f, g). This data suggests that the mechanical behavior of cells is significantly affected by the spatial arrangement of the different cell types in mixed-cell populations.

## Discussion

In this study, we determined that co-culture with different cell types can significantly alter the traction, intercellular stress, and velocity within a monolayer of mammalian epithelial cells. Our findings suggest that interactions with neighboring cells are pivotal in determining mechanical behaviors of cells. This challenges the common assumption that cell mechanics, being largely genetically determined, remain consistent irrespective of interactions with other cell types. Comparable shifts in cell mechanics have been observed at the interface between distinct cell types [48–50]; for example, junction tension increases at compartment boundaries in embryos and at clonal boundaries separating normal and oncogenic cells. Our *in situ* mechanical measurements revealed that the mechanical changes were not localized, but spread throughout the entire monolayer without dependence on the distance to the cell-type boundaries. These observations highlight a collective mechanism that acts over long distances for triggering the mechanical convergence. Investigating the distribution and activity of molecules that control cell mechanics is crucial to further support our findings and shed light on the mechanisms by which interactions between diverse cell types are integrated with genetical regulations to control the mechanics of mixed-cell populations.

The mechanisms underlying the differences between localized changes at the interfaces and more global changes within the entire monolayer remain elusive. One critical factor could be the cell mixing ratio, which was set to 1:1 in this study. Determining the specific cell mixing ratio at which a mixed monolayer begins to behave as a cohesive entity is of interest. In addition, the interface geometry should be considered because it can influence both the local cell mixing ratio and the magnitude of intercellular signaling [51, 52] (see also discussion about the modeling results). Biological tissues may utilize these geometrical parameters to dynamically adjust cell mechanics during tissue development and repair.

Differential cell adhesion has been known to induce cell sorting [15, 16, 41]. However, in our experiments with WT and E-cad KO cells in MDCK-II monolayers, no significant segregation into large domains was observed. To eliminate the potential effects of cell proliferation and jamming, we incubated the cells with a cell cycle inhibitor for 1.5 h. This inhibition of cell proliferation prevented clonal expansion, which may have contributed to domain expansion among cells of the same type. Without cell proliferation, additional mechanisms, such as ephrin-Eph-based repulsion [53], may be required for the efficient sorting of cells in MDCK-II monolayers.

We introduced a simple hydrodynamic model for a binary cell mixture and uncovered that two phases, each with intrinsically distinct self-propelled forces, can behave like a single entity when mixed. We speculate that the force transmission within the cell population aligns the movement of the two phases, leading to a global alteration in the stress field. The well segregation of phases, however, impedes this coherent movement, thus preventing mechanical convergence. As long as the cellular domains remain small, a moderate level of disorder in the polarity and velocity fields has a minor impact on the mechanical convergence in mixed-cell populations. The insights gleaned from this model provide clues to a cell–cell communication mechanism that drives the observed global changes in velocity and stress fields in co-culture experiments.

Although our mixed-phase model suggests a physical mechanism driving the convergence of traction, intercellular stress, and velocity, it does not account for the direction of the shifts in these mechanical quantities. In the WT+RasV12 monolayer, we observed that the values of traction and intercellular stress were intermediate to those in the WT and RasV12 monolayers, while the velocity exceeded that seen in single-cell experiments. Conversely, in the WT+E-cad KO monolayer, the velocity fell between those in the WT and E-cad KO monolayers, yet the traction and intercellular stress were lower than those in single-cell experiments. These variations underscore the complex interactions between different cell types and highlight the need for further investigation to fully understand the underlying dynamics governing these mechanical shifts.

In conclusion, this study highlights the importance of *in situ* mechanical measurements in mixed-cell populations for understanding the mechanics of multicellular systems. As the available mechanical tools continue to expand [25, 26], we anticipate that various mechanical quantities will be directly quantified in mixed-cell populations. This will enable future studies to explore the intricate interplay between the genetics and mechanics in heterotypic tissue environments.

## Materials and methods

### Cell lines

MDCK-II and MDCK cells were used in this study. WT MDCK-II cells and E-cad KO MDCK-II cells were a gift from Tetsuhisa Otani. WT-GFP MDCK-II cells were generated by transfecting MDCK-II cells with pCANw-EGFP (CAG-GFP-IRES-neo) [54] using Lipofectamine LTX followed by selection with 400 ${\upmu }$g/ml G418. The E-cad KO MDCK-II cell line was established in [42]. The MDCK cell lines used are described in [33].

### Cell culture

MDCK-II cells were cultured in Dulbecco’s modified Eagle’s medium (DMEM) (Gibco) supplemented with 10% fetal bovine serum (FBS) (Biowest) and 1% penicillin/streptomycin (Gibco) at 37°C and 5% CO_2_. WT-GFP MDCK-II cells were maintained with 500 ${\upmu }$g/ml of G418 (Geneticin) (Gibco). MDCK cells were cultured in DMEM (Wako) supplemented with 10% FBS (Sigma-Aldrich), 1% penicillin/streptomycin (Life Technologies), and 1% GlutaMax (Life Technologies) at 37°C and 5% CO_2_. MDCK-GCaMP6s cells and MDCK-pTRE3G Myc-RasV12 cells were maintained with 80 ${\upmu }$g/ml of G418 (Geneticin) (Gibco) and 0.5 ${\upmu }$g/ml blasticidin (Invitrogen), respectively [55, 56]. Mycoplasma contamination was regularly tested in all cell lines using a MycoAlert Mycoplasma Detection Kit (Lonza).

### Preparation of soft substrate

Experiments were performed in a 35-mm glass-bottom dish (Iwaki). The glass bottom of each dish was covered with a soft gel created by mixing parts A and B of the silicone elastomer DOWSIL CY 52-276 (Dow Corning). For experiments with MDCK-II cells, a soft substrate with a Young’s modulus of 15 kPa was obtained by mixing parts A and B in a 1:1 ratio, whereas a soft substrate with a Young’s modulus of 3 kPa (A:B ratio of 6:5) was used for experiments with MDCK cells [57]. Soft substrate dishes were then covered with 200-nm diameter, fluorescent beads (red for MDCK-II experiments and dark red for MDCK experiments) (Invitrogen) to track the traction exerted by the cells over time.

### Live cell imaging

In the experiments with MDCK-II cells, the cells were treated with Mitomycin C (Sigma-Aldrich), a cell cycle inhibitor, at a concentration of 10 ${\upmu }$g/ml for 1.5 h, 24 h before cell seeding [58]. On the day of cell seeding, a microprinting technique was performed to stamp fibronectin (Wako) onto a soft substrate covered with beads [39]. The fibronectin was used at a concentration of 50 ${\upmu }$g/ml and was stamped in the shape of 1.8 mm diameter circles. Pluronics F-127 (Sigma-Aldrich) at a concentration of 2% was used to prevent the cells from adhering outside of the circles. After 1 h of incubation, Pluronics F-127 was rinsed with PBS (Gibco) and MDCK-II culture medium. In experiments involving single-cell-type cultures, cells were directly plated on a stamped soft substrate-covered dish, whereas in the case of mixed-cell-type experiments, cells from each cell line were first transferred to a vial in the desired proportion and then seeded on the dish. After incubating the cells at 37°C with 5% CO_2_ for 1 h, the cells were rinsed with PBS and culture media until no floating cells remained. Image acquisition began 24 h later, following a change of the culture media. Images were captured using an inverted confocal spinning disk microscope (Olympus IX83 combined with Yokogawa CSU-W1) equipped with an iXon3 888 EMCCD camera (Andor), an Olympus 20x/NA0.45 LUCPLFLN PH dry objective, and a temperature control chamber (TOKAI HIT), using IQ 2.9.1 software (Andor). A number of 3x3 fields of view are required to capture a monolayer of cells on a 1.8 mm diameter circle. Images were captured at 37°C and 5% CO_2_ for 24 h at 12 min intervals.

Soft substrate dishes used for experiments with MDCK cells were coated with 0.3 mg/ml collagen at 4°C for 16 h and subsequently washed with PBS [33]. MDCK cells were incubated in Leibovitz’s medium (L-15) (Gibco) supplemented with 10% FBS. MDCK-GCaMP6s cells were mix-cultured with MDCK or MDCK-pTRE3G Myc-RasV12 cells at a ratio of 1:1 and plated on a soft substrate dish. To prepare a sample of RasV12 alone, MDCK-pTRE3G Myc-RasV12 cells were pre-stained with CellTracker Dye CMFDA (green) (Life Technology) before mixing with unstained MDCK-pTRE3G Myc-RasV12 cells. The mixture of cells was incubated for 12–16 h until a monolayer was formed, followed by the treatment of 1 ${\upmu }$g/ml doxycycline (Sigma-Aldrich) for 6 h to induce the expression of RasV12. Images were captured using an inverted confocal microscope (Nikon A1 HD25) equipped with a Nikon 25x/NA1.05 PLAN APO silicon oil-immersion objective and a temperature control chamber (TOKAI HIT). Images were captured at 37°C for 4 h at 10 min intervals, starting at 6 h after the induction of RasV12.

### Image analysis

*Movie creation*

For experiments with MDCK-II cells, images were re-ordered using ImageJ/Fiji to construct individual movies of phase contrast, GFP, and red fluorescent beads. For phase contrast and bead movies, the focused slice at each time frame was selected, whereas the z-slices of the GFP movies were projected to increase the signal-to-noise ratio. Images at different x- and y-positions were stitched together using the Fiji Grid/Collection stitching plugin [59] to cover 1.8-mm-diameter circles. Finally, for both experiments with MDCK and MDCK-II cells, the bead movies were stabilized to remove any shifts in the x- and y-directions.

*Mask creation*

A custom-made ImageJ/Fiji macro was used to create black and white masks to isolate the monolayer of cells from the outside of the monolayer (made from the phase contrast movies and called cell masks), and to separate the two populations of cells within a mixed-culture experiment using the GFP signal of one of the cell lines (made from the GFP fluorescence movies and called GFP masks) (Fig. 1). We also employed black and white masks to calculate mechanical values within smaller ROIs, thereby excluding any values outside the chosen area from the calculation.

*Velocity fields*

Velocity fields were calculated using the MatPIV toolbox for MATLAB (MathWorks). This allowed us to perform Particle Image Velocimetry (PIV) on the phase-contrast movies between consecutive frames using interrogation window sizes of approximately 20 ${\upmu }$m for MDCK cells experiments, and 40 ${\upmu }$m for MDCK-II cells experiments, with 50% overlap in both cases.

*Quantification of calcium sparks*

MorpholibJ, a Fiji plugin, was used to segment the inverted bright field images and label the outer contours of individual MDCK cells. If necessary, the segmented images were corrected manually. In addition, a GFP mask was generated to identify cells expressing GCaMP6s. Each GFP-positive cell was tracked over time using the Trackmate plugin in Fiji. For the analysis, we excluded cells located at the edge of the field of view and those that could not be tracked over the entire duration of the experiment. Calcium sparks were defined as events in which the intensity of GCaMP in a cell exceeded the mean GFP intensity by more than five standard deviations [33]. Physical quantities (traction and isotropic and deviatoric stress values) were then averaged at each time frame in a circle of a chosen radius around the centroid of the selected cells and plotted over time for each cell (Figs. 3 and A2).

### Traction force microscopy (TFM) and intercellular stress calculation

*Traction force microscopy*

PIV was used to track the bead displacements with interrogation windows of 16 and 10 ${\upmu }$m used for the experiments performed, respectively, with MDCK and MDCK-II cells, and an overlap of 75%. Subsequently, the ImageJ/Fiji FTTC plugin [60] with a regularization factor of 8e-11 was used to obtain the tractions $\overrightarrow{T}(x,y,t)~=~[T_x(x,y,t),T_y(x,y,t)]$ from the displacement fields.

*Stress tensor calculation*

Intercellular stress $\sigma$ was obtained from the traction $\overrightarrow{T}$ through the relationship div $\sigma = \overrightarrow{T}$ using Bayesian inversion stress microscopy (BISM) [27]. A two-dimensional symmetrical stress tensor $$\sigma = \bigl ( {\begin{matrix} \sigma _{xx} &{} \sigma _{xy} \\ \sigma _{xy} &{} \sigma _{yy} \end{matrix}} \bigr )$$ was calculated in each interrogation window of the PIV grid used for TFM. From each stress tensor $\sigma$, we calculated the isotropic stress $\sigma _{iso}$ which is the trace of the tensor as $\sigma _{iso} = \frac{\sigma _{xx}+\sigma _{yy}}{2}$. The value $\sigma _{iso}$ is a scalar and the opposite of the pressure inside the tissue, so that $\sigma _{iso} < 0$ means compression in the tissue, and $\sigma _{iso} > 0$ means that the tissue is under tension. The deviatoric stress $\sigma _{dev}$ is a symmetrical and traceless tensor defined as $\sigma _{dev} = \sigma - \sigma _{iso}I$ where *I* is the identity matrix. By applying a linear transformation to the deviatoric stress tensor $\sigma _{dev}$, we determine its eigenvalues, $\lambda _{dev}$ and $-\lambda _{dev}$. Since $\sigma _{dev}$ is a symmetrical and traceless tensor, these eigenvalues are equal in magnitude but opposite in sign. We use the absolute value $|\lambda _{dev}|$ to represent the magnitude of the deviatoric stress.

### Statistics

P values were calculated in MATLAB using *ttest2* (two-sample t test) and *ranksum* (two-sided Wilcoxon rank-sum test).

### Model

*Detailed expression of elastic stress, chemical potential, and molecular field*

Elastic stress tensor $\sigma ^e =\sigma ^p + \sigma ^\phi$ is derived from the free energy as

8 $$\begin{aligned} \sigma ^p_{ij}&= \frac{1}{2}\left( p_iH_j-H_ip_j\right) -\frac{\kappa }{2}\left( p_iH_j+H_ip_j\right) \nonumber \\&\quad - \nabla _i p_k \frac{\partial F}{\partial (\nabla _j p_k)}~, \end{aligned}$$

9 $$\begin{aligned} \sigma ^\phi _{ij}&= \left( {f}- \phi \mu \right) \delta _{ij} -\nabla _i \phi \frac{\partial F}{\partial (\nabla _j \phi )}~. \end{aligned}$$

where *f* is the integrand in Eq. 1. Detailed expressions for chemical potential $\mu$, molecular field ${{\varvec{H}}}$, and elastic stresses $\sigma ^p$ and $\sigma ^\phi$ are as follows.

10 $$\begin{aligned} \mu&= -a(1-\phi ^2)\phi -K_\phi \nabla ^2 \phi \end{aligned}$$

11 $$\begin{aligned} H_i&= -a_p (p_0 - |p|^2)p_i -K_p \nabla ^2 p_i \end{aligned}$$

12 $$\begin{aligned} \sigma ^p_{ij}&= \frac{1}{2}\left( p_iH_j-H_ip_j\right) -\frac{\kappa }{2}\left( p_iH_j+H_ip_j\right) \nonumber \\&\quad - K_p \partial _i p_k \partial _j p_k \end{aligned}$$

13 $$\begin{aligned} \sigma ^\phi _{ij}&= -K_\phi \partial _i \phi \partial _j \phi \end{aligned}$$

In general, the coefficients $\nu , \alpha , \xi$, and $K_p$ can be functions of $\phi$. In this study, we assume differences in self-propelled forces and viscosity between two types of cells (Eqs. 6 and 7), while the other coefficients are constant. For calculating $\mu$, we neglected the contribution from the derivative of the coefficients.

*Parameters*

In our simulation, time and length units were chosen as $10~\textrm{min}$ and $10~\mu \textrm{m}$, respectively. The system size, a $50 \times 50$ square region in the simulation unit, is equivalent to $500 \times 500~\mu \textrm{m}^2$. The simulation duration in Fig. 7d–g corresponds to $600~\textrm{min}$. Characteristic quantities were determined as follows. Intrinsic cell migrating velocities are $\alpha _A \xi ^{-1} = 1.0~\mathrm{\mu m/min}$ and $\alpha _B \xi ^{-1} = 0.5~\mathrm{\mu m/min}$, reflecting the experimental observation in E-cad KO and WT MDCK-II cells (Fig. 6). $(Ma)^{-1} = 10~\textrm{min}$ was set so that $\phi$ maintains the sharp interface during simulations. $(a_p\Gamma )^{-1} = 20~\textrm{min}$ indicates the characteristic timescale for cell polarity formation. The interface width of the two-cell population is given as $\sqrt{K_\phi /2a} = 5~\mu \textrm{m}$. The correlation length for the polar field estimated by $\sqrt{K_p/2a_p}$ is $\sim 20~\mu \textrm{m}$ at $K_p = 1.0$ (Fig. 7a, c) and $\sim 3~\mu \textrm{m}$ at $K_p = 0.02$ (Fig. 7b). The correlation length of the velocity field is estimated by $\sqrt{\nu /\xi } \sim 30~\mu \textrm{m}$. The temporal and spatial correlations of cell velocity are comparable with our experimental observations in MDCK-II cells. $\kappa$ is a non-dimensional parameter and was set to $\kappa = 0.7$ following [45]. Collectively, these parameter values are within reasonable ranges.

*Numerical simulations*

We solved Eqs. (1)–(4) on a $L \times L = 50 \times 50$ square region with periodic boundary conditions. The square region is discretized by triangular mesh, whose size is at most 1.0. The simulation was carried out by a finite element method (FEM) using a FEM solver, FreeFEM++ [61]. Pressure is approximated using the P1 finite element, while other variables are by P2. For solving Eqs. (2) and (3), the Crank–Nicolson scheme was used, while the FreeFEM++ operator **convect()** was applied for calculating the convection term [61]. Time discretization was $\Delta t = 1.0 \times 10^{-2}$.

### Supplementary Information

Below is the link to the electronic supplementary material.

Supplementary file 1 (pdf 735 KB)

Supplementary file 2 (avi 4078 KB)

Supplementary file 3 (avi 68936 KB)

Supplementary file 4 (avi 9885 KB)

Supplementary file 5 (avi 8732 KB)

Supplementary file 6 (avi 7920 KB)

## Data Availability

The authors declare that the data supporting the findings of this study are available within the paper and its Supplementary files. The data are available from the lead contact (Kaoru Sugimura) upon reasonable request. The code used for numerical simulations can be downloaded from https://github.com/IshiharaLab/BinaryActivePolarModel.
