# Supplementary material for: Mechanical convergence in mixed populations of mammalian epithelial cells
Source: Eur Phys J E Soft Matter. 2024 Mar 27;47(3):21. doi: 10.1140/epje/s10189-024-00415-w (PMC10973031; doi:10.1140/epje/s10189-024-00415-w)
Supplement: Supplementary file 1 — (pdf 735 KB) [file 10189_2024_415_MOESM1_ESM.pdf]

Appendix A Supplementary Figures

Figure A1 We varied the size of the ROIs to assess potential size and positional dependencies in the measured mechanical quantities. Given that the patterns employed have a typical length R (either the radius of the circles or the side length of the square fields of view), we performed the quantification of traction, isotropic stress, and deviatoric stress in areas that have typical lengths of $0.95R$ and $\frac{2}{3}R$. In both cases, the results closely resemble those obtained in the whole domains (compare Fig. A1 with Fig. 2 and Fig. 4).

Figure A2 We tracked the traction and intercellular stress over time in regions proximal to the cells producing calcium sparks in Fig. 3. Here, we verified that the size of the area of calculation had no significant effect on the evolution of the mechanical quantities surrounding calcium spark events.

Appendix B Supplementary Table

Table A1 List of parameters and their physical dimensions.

Appendix C Supplementary Videos

Video A1 Intercellular stress in monolayers of WT MDCK cells, RasV12-induced MDCK cells, and mixed-cultures of WT+RasV12 cells.
Overlay of isotropic stress as colormaps with principal stress tensors as elliptical principal axes for WT MDCK cell monolayers (a), RasV12-induced MDCK cell monolayers (b), and mixed-cultures of WT and RasV12-induced cells (c). The stress tensor axes are presented in red when the stress value is positive, and in blue when it is negative. Scale bar: 100 µm.

Video A2 Intercellular stress in monolayers of WT MDCK-II cells, E-cad KO MDCK-II cells, and mixed-cultures of WT+E-cad KO cells.
Overlay of isotropic stress as colormaps with principal stress tensors as elliptical principal axes for WT MDCK-II cell monolayers (a), E-cad KO MDCK-II cell monolayers (b), and mixed-cultures of WT and E-cad KO cells (c). The stress tensor axes are presented in red when the stress value is positive, and in blue when it is negative. Scale bar: 500 µm.

Video A3 Numerical simulation of binary cell mixture shown in Fig. 7a.
The phase field (color scale) and velocity field (blue arrows) at the indicated simulation time. Orange and yellow indicate regions occupied by cells A ($\phi = 1$) and cells B ($\phi = -1$), respectively. Parameter values are described in the legend of Fig. 7.

Video A4 Numerical simulation of binary cell mixture shown in Fig. 7b.
The phase field (color scale) and velocity field (blue arrows) at the indicated simulation time. Orange and yellow indicate regions occupied by cells A ($\phi = 1$) and cells B ($\phi = -1$), respectively. Parameter values are described in the legend of Fig. 7.

Video A5 Numerical simulation of binary cell mixture shown in Fig. 7c.
The phase field (color scale) and velocity field (blue arrows) at the indicated simulation time. Orange and yellow indicate regions occupied by cells A ($\phi = 1$) and cells B ($\phi = -1$), respectively. Parameter values are described in the legend of Fig. 7.

Parameter	Simulation value	Dimension
a	0.1	Pa
K_ϕ	0.05	Pa m^2
a_p	0.1	Pa
K_p	0.02 – 1.0	Pa m^2
M	10.0	Pa^{-1}s^{-1}
Γ	5.0	Pa^{-1}s^{-1}
κ	0.7	dimensionless
ν_A	5.0	Pa s
ν_B	10.0	Pa s
α_A	1.0	Pa m^{-1}
α_B	0.5	Pa m^{-1}
ξ	1.0	Pa s m^{-2}

Table A1: List of parameters and their physical dimensions. Names, values used in simulations, and dimensions are listed for each parameter. The mathematical model is two-dimensional and the integrand in Eq. 1 represents the energy density per area with the physical unit Pa m. In the simulations, time and length units are set as 10 min and 10 μm, respectively. See Materials and Methods for the rationale of the parameter choice.

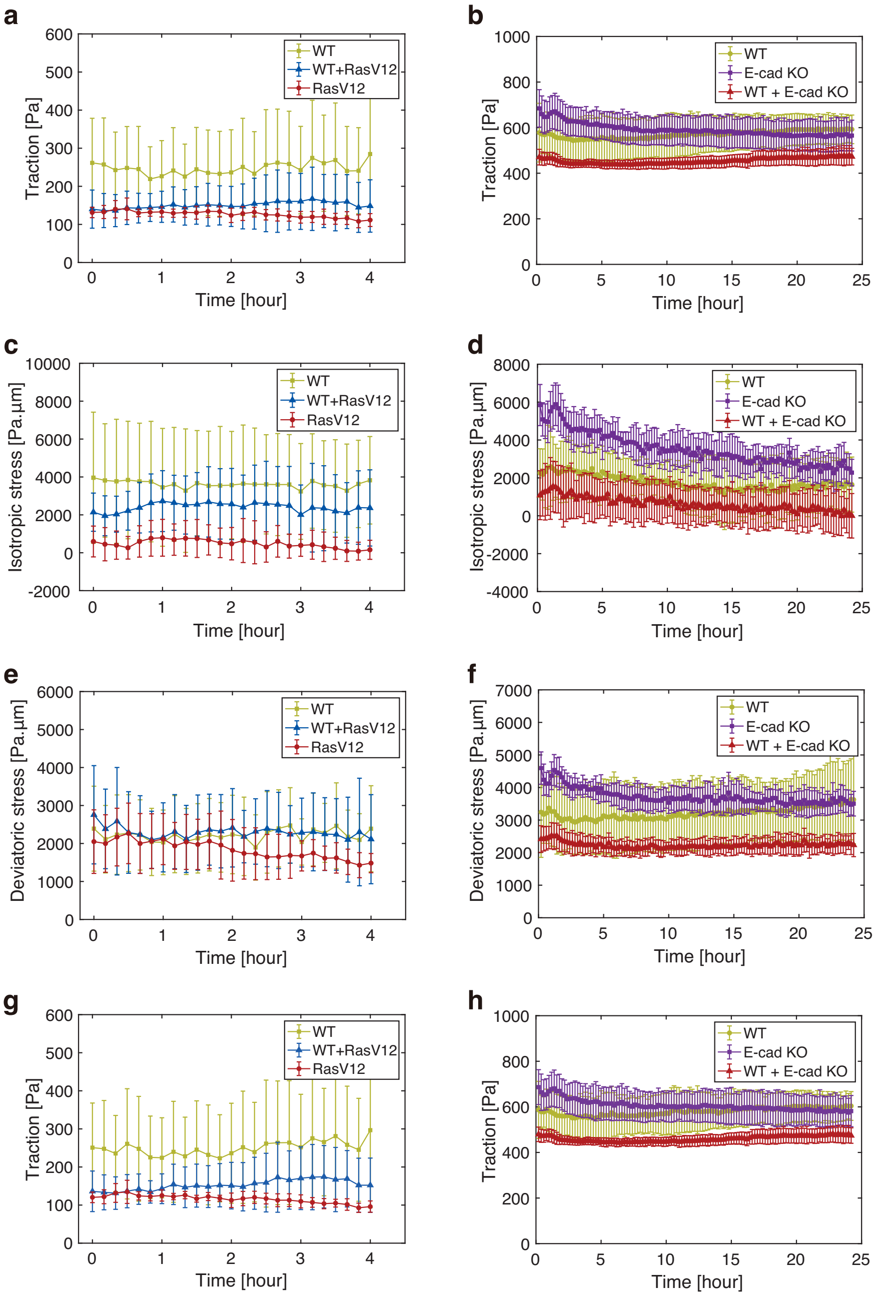

Fig. A1: **Quantification, in areas of typical length** $0.95R$ **and** $\frac{2}{3}R$**, of traction, isotropic stress, and deviatoric stress.** (a, b) Magnitude of the tractions, in areas of typical length $0.95R$, exerted by the cells in a function of time depending on the experiment types in MDCK cells (n = 3 for each plot) (a) and in MDCK-II cells (WT: n = 8, E-cad KO: n = 6, and WT+ E-cad KO: n = 8) (b). In (a), 0 hour corresponds to 6 h after the induction of RasV12 expression. WT represents the co-culture of GCaMP WT and non-tagged WT MDCK cells (light green), WT+RasV12 represents the co-culture of GCaMP WT and Myc-tagged RasV12-induced MDCK cells (blue), and RasV12 represents the co-culture of CMFDA-stained RasV12-induced and unstained RasV12 MDCK cells (red). In (b), 0 hour corresponds to 24 h after the cell seeding. WT represents the WT MDCK-II cells in single-cell colonies (light green), E- cad KO represents the E-cad KO MDCK-II cells in single-cell colonies (purple), and WT+E-cad KO represents the co-culture of WT and E-cad KO MDCK-II cells (red). (c, d) Isotropic stress, in areas of typical length $0.95R$, in a function of time depending on the experiment type in MDCK cells (c) or MDCK-II cells (d), as shown respectively in (a) and (b). (e, f) Magnitude of deviatoric stress, in areas of typical length $0.95R$, in a function of time depending on the experiment type in MDCK cells (e) or MDCK-II cells (f), as shown respectively in (a) and (b). (g, h) Magnitude of the tractions, in areas of typical length $\frac{2}{3}R$, exerted by the cells in a function of time depending on the experiment types in MDCK cells (f) or MDCK-II cells (g), as shown respectively in (a) and (b). Data is presented as the mean ± s.d.

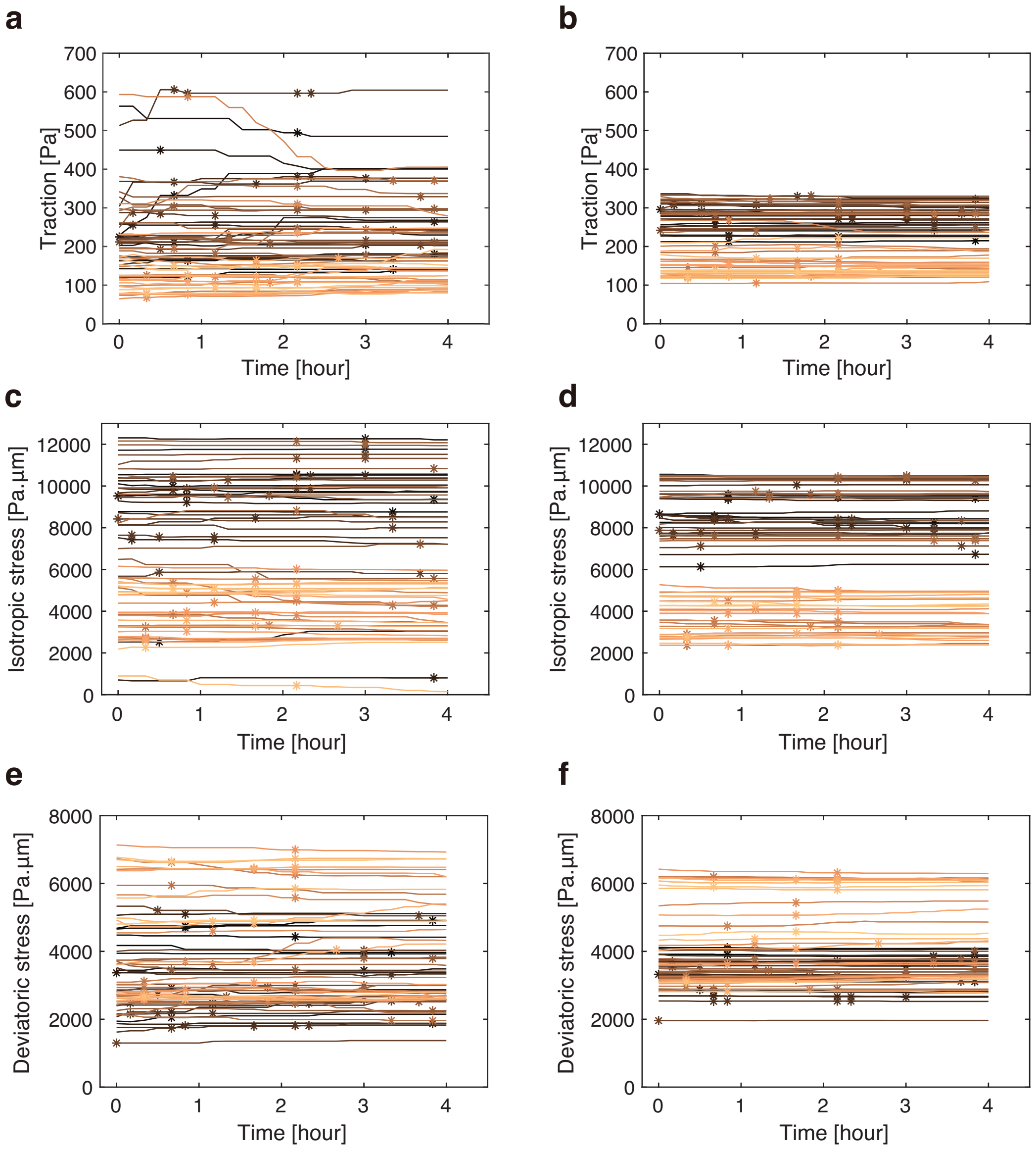

Fig. A2: **Evolution of mechanical quantities calculated in areas of different sizes surrounding calcium spark events.** Quantification of traction (a, b), isotropic stress (c, d), and deviatoric stress (e, f) values in an area around cells producing a spark over the time of the experiment. In (a), (c), and (e), the radius of the area around the sparking cells used to calculate the averaged quantities is 29 µm; and in (b), (d), and (f), the radius is 102 µm. The horizontal axis indicates time after the start of image acquisition, with the 0-hour mark corresponding to 6 h after induction of RasV12 expression. Lines represent data for each ROI, and calcium spark occurrences are indicated by stars.
